# Origin of 3 Rabid Terrestrial Animals in Raccoon Rabies Virus–Free Zone, Long Island, New York, USA, 2016–2017

**DOI:** 10.3201/eid2606.191700

**Published:** 2020-06

**Authors:** Scott Brunt, Heather Solomon, Hilaire Leavitt, Erica Lasek-Nesselquist, Pascal LaPierre, Matt Shudt, Laura Bigler, Navjot Singh, April D. Davis

**Affiliations:** New York State Department of Health, Albany, New York, USA (S. Brunt, H. Solomon, E. Lasek-Nesselquist, P. LaPierre, M. Shudt, N. Singh, A.D. Davis);; Connecticut Department of Health, Rocky Hill, Connecticut, USA (H. Leavitt);; Cornell University, Ithaca, New York, USA (L. Bigler)

**Keywords:** rabies, raccoon variant, whole-genome sequencing, United States, phylogeny, epidemiology, zoonoses, oral rabies vaccination, wildlife surveillance, phylogenetic analyses, Long Island, New York, rabies-free zone, viruses

## Abstract

During 2016–2017, three rabid terrestrial animals were discovered in the raccoon rabies virus–free zone of Long Island, New York, USA. Whole-genome sequencing and phylogenetic analyses revealed the likely origins of the viruses, enabling the rabies outbreak response (often costly and time-consuming) to be done less expensively and more efficiently.

Rabies has been endemic to the eastern United States in raccoons (*Procyon lotor*) since 1960 and endemic to New York, USA, since the 1990s (*1*). The spread of rabies virus from raccoons in the mid-Atlantic states to species in New York has been reconstructed with spatiogenetic and phylogenic analyses (*2*). Despite this spread, a few locations in the region, including Suffolk and Nassau Counties on Long Island, New York, have been considered to be rabies free since 2011 (*3*). Maintaining these areas as low risk for rabies substantially decreases the likelihood of human and animal exposure to rabies virus and dramatically reduces the expenses paid for postexposure rabies virus treatments and rabies control (*4*).

To identify any breaches in the elimination zone, Nassau and Suffolk County health departments, veterinarians, and wildlife control officers routinely submit animals with clinical signs compatible with rabies for rabies virus testing to the New York State Department of Health Rabies Laboratory (Slingerlands, New York, USA). Before 2016, rabies had not been reported in Suffolk County since 2009 or in Nassau County since 2007 (Figure 1) (*5*). During 2016–2017, rabies reappeared in Nassau and Suffolk Counties in 3 terrestrial animals: a raccoon (*Procyon lotor*), a river otter (*Lontra canadensis*), and a domestic cat (*Felis catus*). Here, we describe our efforts to identify the origins of these rabid animals and our contingency plans to eliminate further cases and restore the rabies virus–free zone status.

**Figure 1:**
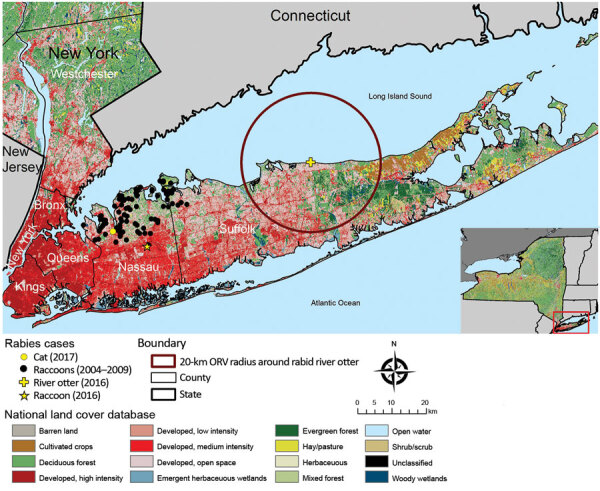
Locations of rabid raccoon, river otter, and cat in raccoon rabies virus–free zone, Nassau and Suffolk Counties, Long Island, New York, USA, 2016–2017. Locations of rabid raccoons found in these counties before they became raccoon rabies virus free are also indicated. The 20-km radius originally proposed for the distribution of ORV if rabies virus had become reestablished in raccoon rabies virus–free zone is indicated. Inset indicates location of Long Island in New York. ORV, oral rabies vaccine.

## The Study

In March 2016, a rabid raccoon with neurologic signs was trapped in Hicksville in Nassau County. Real-time reverse transcription PCR (*6*) and Sanger sequencing demonstrated that the animal was infected with a raccoon rabies virus variant. A phylogenetic analysis of the full nucleoprotein and partial glycoprotein gene sequences did not provide us the resolution required for us to confidently identify where the virus had originated. At the time of this original analysis, whole-genome sequencing (WGS) was not available in our laboratory. We initiated enhanced rabies surveillance for ≈12 months in Babylon and Huntington of Nassau County to determine if rabies virus was circulating locally by increasing the number of wild animals submitted for testing via trapping, roadkill collection, and animal control activities, but no animals were positive for rabies.

In December 2016, an employee of the New York State Department of Environmental Conservation (Albany, New York, USA) found a river otter on Sound Beach on the North Shore of Suffolk County; the otter was submitted to the Rabies Laboratory of the New York State Department of Health in February 2017. After the otter was determined to be rabies virus positive, our goal was to ascertain by WGS and sequence analysis where the otter was exposed, which would help us determine which supplementary contingency actions were required.

In November 2017, a cat with neurologic signs that bit a veterinary staff member in Nassau County was submitted for testing. After determining this cat was positive for rabies virus, we included this case in the study.

When the raccoon was originally discovered on Long Island, we had contacted multiple state and federal agencies to discuss contingency plans for containing a possible rabies outbreak. We agreed to limit the contingency actions at that juncture to 12 months’ enhanced surveillance, public health alerts to educate citizens, and reminders about free rabies virus vaccine clinics for domestic animals. Only in the event that other rabid animals were discovered during this time would wildlife trap, vaccinate, and release programs be implemented and oral rabies virus vaccine baits be distributed (*4*,*5*,*7*). These vaccination programs can be expensive, so they are used conservatively as last resort efforts. The cost of the raccoon rabies virus elimination program that occurred on Long Island during 2006–2010 was ≈$2.6 million (*4*).

The contingency plan to respond to the rabid river otter was initially to deploy oral rabies vaccine around a 20-km radius of Sound Beach Long Island, covering nearly all of central Suffolk County (Figure 1). Costs would have exceeded $200,000 as 120,000 baits would need to have been spread by foot, vehicle, and aircraft over a 636.6-km^2^ area during spring 2017. Because Wadsworth Center Sequencing Core (Slingerlands, New York, USA) charged ≈$250/sample for WGS, we were able to save time and money by sampling animals and ruling out some areas as having local transmission and deescalating the initial plan. Applying WGS to priority samples has previously been shown to be a viable strategy for public health laboratories to use in epidemiologic applications (*8*).

**Figure 2:**
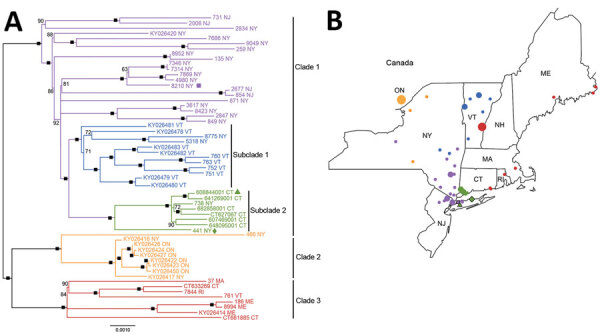
Maximum-likelihood whole-genome phylogeny and geographic location of rabies virus variants, northeastern United States and Canada, 2016–2017, including the rabid raccoon (green triangle), river otter (green diamond), and cat (purple square) found in raccoon rabies virus–free zones, Nassau and Suffolk Counties, Long Island, New York, USA . A) Midpoint-rooted, maximum-likelihood sequence analysis depicts the relationships among variants collected from New York, New Jersey, Massachusetts, Connecticut, Rhode Island, Vermont, and Maine, USA, and Ontario, Canada. Black boxes indicate nodes with >95% bootstrap support. Bootstrap support <50% is not shown. Scale bar indicates nucleotide substitutions per site. B) Location (by county) of virus isolation. Colors represent the major clades or subclades depicted in tree, and the size of symbols is proportional to the number of rabies samples isolated in that county. ON, Ontario.

Using phylogenetic analysis (Table; Figure 2), we found that the raccoon in Hicksville was infected with a rabies virus variant consistent with those found in southwestern Connecticut. Considering the absence of local rabid wildlife with a similar rabies virus variant, we hypothesized that this raccoon was either accidentally or purposefully translocated into Long Island by human activity. Such translocation events were previously demonstrated with the linkage of an outbreak in Canada to a clade of raccoon rabies virus from upstate and central New York (*9*) and the spread of raccoon rabies virus up the East Coast of the United States (*2*,*8*). Furthermore, although the range of the average raccoon is highly dependent on many factors, raccoon ranges have been established to rarely exceed 4 km (2.5 miles) (*10*,*11*), well short of the 56-km (35-mile) distance from the Connecticut panhandle to Hicksville, New York.

**Table:** Information on rabies viruses used for phylogenetic analysis of viruses isolated from rabid animals found in raccoon rabies virus–free zone, Nassau and Suffolk Counties, Long Island, New York, USA, 2016–2017*

Laboratory ID no.	Collection date	Species	Location	Clade	GenBank accession no.
731-18	2018 Feb 5	Raccoon	Denville, NJ, USA	1	MN418142
2006-18	2018 Apr 23	Raccoon	Lincoln Park, NJ, USA	1	MN418156
2834-17	2017 Jun 16	Red Fox	New City, NY, USA	1	MN418149
KY026420	2004 Jun 21	Raccoon	Genoa, NY, USA	1	†
7686-17	2017 Oct 6	Skunk	Troy, NY, USA	1	MN418168
9049-17	2017 Dec 16	Raccoon	Hudson, NY, USA	1	MN418162
259-17	2017 Jan 23	Raccoon	Clinton, NY, USA	1	MN418178
8952-08	2008 Nov 26	Raccoon	Lloyd Harbor, NY, USA	1	MN418143
135-09	2009 Jan 8	Raccoon	Huntington, NY, USA	1	MN418164
7346-17	2017 Sep 21	Raccoon	Bronx, NY, USA	1	MN418174
7314-17	2017 Sep 20	Raccoon	Bronx, NY, USA	1	MN418157
7869-17	2017 Aug 18	Raccoon	Tarrytown, NY, USA	1	MN418153
4980-17	2017 Aug 8	Gray Fox	Mahopac, NY, USA	1	MN418183
**8210-17**	**2017 Nov 4**	**Cat**	**Sea Cliff, NY, USA**	**1**	**MN418154**
2677-18	2018 May 26	Raccoon	Midland Park, NJ, USA	1	MN418169
854-18	2018 Feb 16	Raccoon	Upper Saddle River, NJ, USA	1	MN418171
871-17	2017 Mar 3	Raccoon	New Paltz, NY, USA	1	MN418145
3617-17	2016 Jul 12	Raccoon	Cornwallville, NY, USA	1	MN418152
8423-17	2017 Nov 11	Skunk	Accord, NY, USA	1	MN418172
2847-17	2017 Jun 6	Gray Fox	Monticello, NY, USA	1	MN418158
849-17	2017 Mar 19	Raccoon	Slate Hill, NY, USA	1	MN418167
KY026481	2008 Sep 18	Raccoon	Charlotte, VT, USA	1 subclade 1	†
KY026478	2006 Nov 16	Raccoon	Stowe, VT, USA	1 subclade 1	†
8775-17	2017 Dec 3	Raccoon	Westerlo, NY, USA	1 subclade 1	MN418144
5318-17	2017 Aug 11	Raccoon	Cobleskill, NY, USA	1 subclade 1	MN418147
KY026483	2011 Oct 23	Skunk	Walden, VT, USA	1 subclade 1	†
KY026482	2008 Sep 28	Raccoon	Stowe, VT, USA	1 subclade 1	†
760-18	2017 Aug 29	Raccoon	Brownsville, VT, USA	1 subclade 1	MN418146
763-18	2017 Jun 20	Gray Fox	Arlington, VT, USA	1 subclade 1	MN418177
752-18	2017 Apr 21	Raccoon	Vergennes, VT, USA	1 subclade 1	MN418175
751-18	2017 Mar 3	Red Fox	Bristol, VT, USA	1 subclade 1	MN418165
KY026479	2006 Dec 5	Skunk	Bethel, VT, USA	1 subclade 1	†
KY026480	2007 Dec 10	Raccoon	Braintree, VT, USA	1 subclade 1	†
CT608844	2016 Jul 6	Raccoon	Ridgefield, CT, USA	1 subclade 2	MN418151
CT641269	2016 Nov 15	Raccoon	Ridgefield, CT, USA	1 subclade 2	MN418163
**738-16**	**2016 Mar 3**	**Raccoon**	**Hicksville, NY, USA**	**1 subclade 2**	**MN418159**
CT682858	2017 May 1	Raccoon	Weston, CT, USA	1 subclade 2	MN418160
CT627067	2016 Feb 1	Raccoon	Fairfield, CT, USA	1 subclade 2	MN418182
CT607469	2016 Jun 30	Raccoon	Bridgeport, CT, USA	1 subclade 2	MN418155
CT648095	2016 Dec 14	Raccoon	Weston, CT, USA	1 subclade 2	MN418181
**441-17**	**2016 Dec 14**	**Otter**	**Sound Beach, NY, USA**	**1 subclade 2**	**MN418161**
466-17	2017 Feb 15	Raccoon	Greene, NY, USA	2	MN418176
KY026416	1995 Dec 22	Raccoon	Hounsfield, NY, USA	2	†
KY026426	2000 Jan 12	Raccoon	Wolfe Island, Ontario, Canada	2	†
KY026424	1999 Dec 10	Raccoon	Wolfe Island, Ontario, Canada	2	†
KY026427	2000 Jan 13	Raccoon	Wolfe Island, Ontario, Canada	2	†
KY026422	1999 Jul 14	Raccoon	Prescott, Ontario, Canada	2	†
KY026423	1999 Sep 17	Raccoon	Oxford Station, Ontario, Canada	2	†
KY026450	2001 Apr 18	Raccoon	Leeds, Ontario, Canada	2	†
KY026417	1998 Nov 9	Raccoon	Canton, NY, USA	2	†
37-18	2018 Jan 3	Raccoon	Plymouth, MA, USA	3	MN418173
CT633269	2017 Oct 1	Skunk	Groton, CT, USA	3	MN418148
7844-17	2017 Oct 12	Cat	Providence, RI, USA	3	MN418170
761-18	2017 Aug 19	Raccoon	Woodstock, VT, USA	3	MN418179
186-18	2017 Dec 6	Skunk	Cutler, ME, USA	3	MN418180
8994-17	2017 Dec 15	Raccoon	Washington, ME, USA	3	MN418184
KY026414	2013 May 21	Raccoon	Waldo, ME, USA	3	†
CT661885	2016 Feb 1	Raccoon	New London, CT, USA	3	MN418150

*Bold indicates rabies virus isolates that were found in the rabies virus–free zone. ID, identification. †Previously deposited in GenBank (*8*).

Our phylogenetic analysis could not be used to conclusively determine where the rabies virus variant found in the river otter circulates, although the clade from Connecticut demonstrated the highest sequence similarity. Otters are found on the South Shores of Connecticut, Massachusetts, and Rhode Island and the North Shore of Long Island (*12*). They can swim >32 km (20 miles) a day and occupy territories of considerable size (*13*). When found, the otter was extremely decomposed to the extent of being borderline untestable. We deduced that the otter was likely from coastal or even inland Connecticut on the basis of phylogenetic data, tissue quality, and species behavior and habitat, but we do not know for certain. Additional sampling from Connecticut could potentially result in the virus from the otter being nested definitively within clade 1 subclade 2.

The rabid otter and raccoon most likely represent 2 separate incursion events, evidenced by the phylogenetic analysis. Because enhanced surveillance did not lead to the discovery of additional rabid animals during January 2017–December 2019 in the entirety of Suffolk and Nassau Counties, we can confidently stipulate that these were isolated events. Although we cannot state with conviction that no other animals became infected, these incursions appear to be short lived and self-limiting.

The rabid cat from November 2017 was briefly considered a continuation of this tentative Long Island rabies outbreak, but after several conversations with public health authorities and the pet owner, this cat was determined to have been unvaccinated and adopted ≈80 km (50 miles) away in Westchester County. Phylogenetic analysis confirmed the virus variant was consistent with those found in downstate New York (clade 1). Although international pet adoption often garners headlines, domestic animal adoption (*14*) and incidental rabies translocation remains a constant threat that can jeopardize the results of multimillion-dollar rabies elimination programs*.*

## Conclusions

Using WGS of an assortment of viruses, we revealed the likely origins of 3 raccoon rabies virus variants from 3 animals that had unexpectedly broken into a rabies-free zone. Through intensified enhanced rabies surveillance and WGS, an expensive contingency plan to distribute baits on Long Island ultimately became unnecessary and was not implemented. This study demonstrates the utility of WGS and phylogenetics as part of a multifaceted rabies investigation with tangible real-world consequences.
